# A Leak in the Plumbing: Bladder Rupture in Augmentation Cystoplasty

**DOI:** 10.51894/001c.144629

**Published:** 2025-09-30

**Authors:** Karthik Ranganathan

**Affiliations:** 1 Department of Urology U of M Health Sparrow

## 114

### INTRODUCTION

Augmentation cystoplasty is a method of increasing the compliance of a poorly functioning bladder. It is utilized in severe neurogenic bladder cases with intractable urge incontinence to allow a better quality of life. Often, patients will need to perform routine intermittent straight catheterization (ISC) to avoid bladder distention and upper urinary tract damage. We present a case of decreased frequency of ISC presenting as sepsis due to bladder rupture.

### CASE DESCRIPTION

A 43 year old female presented with respiratory distress in the emergency department requiring intubation for airway protection. She was later admitted to the intensive care unit for sepsis. Urology was consulted for complex urologic history of severe neurogenic bladder secondary to quadriplegia that was previously treated with augmentation cystoplasty. CT abdomen/pelvis on admission showed new diffuse ascites and recent ISC frequency was unclear, prompting an emergent CT cystogram. CT cystogram was positive for intraperitoneal bladder rupture. Patient was taken to the operative room for bladder repair with two defects found near the junction of bladder augment and native bladder.

### DISCUSSION/CONCLUSIONS

Bladder rupture is a serious consequence of increased intravesical bladder pressure. Typically, cases are seen after traumatic etiology. A high index of suspicion should be maintained in those with reconstructive bladder surgery with newfound intra-abdominal fluid. CT cystogram is routine imaging for bladder trauma especially to distinguish extraperitoneal vs intraperitoneal bladder rupture, with the latter requiring operative intervention. Emergent repair is required to avoid peritonitis, renal failure and further progression of sepsis. Bladder reconstructive procedures restore quality of life in severe, intractable neurogenic bladder patients, but require vigilant care to preserve anatomy.

